# Association between Gustave Roussy Immune Score and delay in discharge among children and adolescents with mycoplasma pneumoniae pneumonia: a retrospective cohort study

**DOI:** 10.3389/fped.2025.1564217

**Published:** 2025-10-03

**Authors:** Xu Zhou, Baoqing Zhang, Yanning Li, Xingyou Zhao

**Affiliations:** ^1^ Department of Pediatrics, Affiliated Hospital of Shandong University of Traditional Chinese Medicine, Jinan, Shandong, China; ^2^ First College of Clinical Medicine, Shandong University of Traditional Chinese Medicine, Jinan, Shandong, China

**Keywords:** Gustave Roussy Immune Score, neutrophil-to-lymphocyte ratio, lactic dehydrogenase, albumin, mycoplasma pneumoniae pneumonia, length of stay

## Abstract

**Background:**

Mycoplasma pneumoniae pneumonia (MPP) is one of the leading causes of hospitalization and death in children. Epidemiological studies have confirmed that single blood indicators such as the neutrophil-to-lymphocyte ratio (NLR), lactic dehydrogenase (LDH), and albumin (Alb) levels are associated with the prognosis and length of stay (LOS) of patients with MPP. In this study, we aim to evaluate the association of the GRIm score with the LOS and compare its strength of association with that of its individual components.

**Methods:**

We recruited 786 children with MPP who were admitted to the Affiliated Hospital of Shandong University of Traditional Chinese Medicine during the period between 2014 and 2021. The end of follow-up was discharge. Univariate and multivariate logistics regression models were used to explore the association between the NLR, LDH, Alb as well as the GRIm score with the LOS among our cohort, with odd ratios (ORs) and 95% confidence intervals (CIs). The receiver operating characteristic (ROC) curve, the net reclassification improvement (NRI) index, and the integrated discrimination improvement (IDI) index were used to compare the predictive efficacy of the GRIm score with the NLR and LDH and in predicting the LOS among children and adolescents with MPP.

**Results:**

After the application of the inclusion and exclusion criteria, 389 eligible children and adolescents with MPP were included, with a mean age of 5.21 (±2.59 years). After adjusting for covariates, we found that LDH and the GRIm score were positivity associated with the LOS (LDH: *β* = 0.40, 95% CI: 0.00–0.80; GRIm score: *β* = 0.33, 95% CI: 0.07–0.58). The high GRIm score was associated with an LOS ≥7 or 11 days (≥7 days: OR = 0.635, 95% CI: 0.585–0.685; ≥ 11 days: OR = 0.622, 95% CI: 0.543–0.700). The area under the curve (AUC), NRI, and IDI all showed that the GRIm score had excellent ability in predicting the LOS of children with MPP compared with single indicators such as LDH, the NLR, and Alb.

**Conclusion:**

The GRIm score showed a significant association with the prolonged LOS in children with MPP, and the strength of this association was higher than its individual components. However, these findings still need to be confirmed by further large multicenter prospective cohort studies.

## Introduction

Mycoplasma pneumonia (MP) is a prokaryotic organism without a cell wall structure that lies between bacteria and viruses (1). Like most respiratory infections, MP is transmitted through droplets produced by talking, sneezing, etc. (2). Worldwide, pneumonia caused by MP accounts for approximately 10%–40% of hospitalized community-acquired pneumonia (CAP) among children (3, 4). Mycoplasma pneumoniae pneumonia (MPP) is a self-limited disease with a generally favorable prognosis; however, patients of all ages may develop life-threatening severe CAP or extrapulmonary complications, leading to prolonged hospitalization and increased disease burden (5). Hence, early identification of high-risk patients who may experience delayed discharge and provision of appropriate and targeted interventions are of great significance for improving the prognosis of patients with MPP and for the rational use of medical resources.

MPP is a pathogen that infects the respiratory system by attaching to respiratory epithelial cells, releasing a variety of cytotoxins, and destroying the integrity of the airway mucosa (6). The pathogenic mechanisms of MPP are complex, and the most likely mechanisms include adhesion damage, immune escape, and damage mediated by inflammatory factors (4, 7, 8). The neutrophil-to-lymphocyte ratio (NLR) is a potential biomarker that reflects the body's immune balance and systemic inflammatory response. In recent years, studies have reported that it is associated with the severity of the disease and the length of stay (LOS) in children with MPP (9). Lactate dehydrogenase (LDH) is a key enzyme in lactate metabolism. It connects the metabolism of various immune cells in the tumor microenvironment, activates signal transduction pathways, and regulates immune responses (10). LDH levels are gradually being used to predict the risk and prognosis of children with MPP (11, 12). Albumin (Alb) is an important protein in human plasma synthesized by the liver and plays a key role in maintaining plasma osmotic pressure, transporting and storing nutrients, etc. (13). Zamberlan et al. (14) reported that low Alb levels were associated with a long LOS among children and adolescents with COVID-19. The genome of MP is very small and the biosynthetic and metabolic capacity is rather limited, so the nutrients required for the survival of MP are mainly taken up from the outside. The adhesion and invasion of MPs cause disorders in the carbohydrate metabolism, amino acid intake, and protein synthesis of host cells, leading to nutrient depletion (6). In addition, Alb is an acute-phase reactant, and low levels of Alb may represent increased persistent inflammation in patients with MPP (15). Cheng et al. (16) reported that low Alb levels were independently associated with MPP, and Alb, combined with the neutrophil ratio, LDH, and high fever, can help achieve early prediction of refractory MPP.

While these individual biomarkers provide valuable insights, their interpretation in isolation presents limitations. Each marker reflects a distinct aspect of the host response (inflammation, cellular damage, nutritional/immunological reserve), and their values can be influenced by confounding factors unrelated to MPP severity. Clinically, relying on a single marker may lead to a lack of sufficient specificity or sensitivity for robust risk prediction. Furthermore, the simultaneous assessment and integration of multiple disparate laboratory values (NLR, LDH, and Alb) to form a unified clinical impression can be complex and subjective, potentially hindering rapid and standardized risk stratification at the point of care, particularly in busy pediatric settings. The Gustave Roussy Immune Score (GRIm score), originally developed for prognostic stratification in oncology (17), offers a potential solution to these challenges. It synthesizes three readily available laboratory parameters—NLR, LDH, and serum Alb—into a single, objective, and standardized composite score. By integrating markers reflecting inflammation intensity (NLR), tissue injury (LDH), and nutritional/immunological status (Alb), the GRIm score provides a more holistic snapshot of the underlying pathophysiological state of a patient. Its utility in predicting outcomes in cancer suggests that such integrated scoring systems may outperform individual markers by capturing synergistic or additive effects.

However, the prognostic value of the GRIm score, particularly its ability to predict hospital LOS, remains unexplored in pediatric MPP. Given the significant burden of prolonged hospitalization in children with MPP, there is a critical need for tools enabling early identification of high-risk patients who might benefit from intensified monitoring or targeted interventions. Therefore, this study aimed: (1) to investigate the association between the admission GRIm score and the LOS in children hospitalized with MPP; and crucially, (2) to determine whether this association is stronger than that of its individual components (NLR, LDH, and Alb) alone, as measured by established metrics like the area under the receiver operating characteristic (ROC), net reclassification improvement (NRI), and integrated discrimination improvement (IDI). We hypothesized that the integrated GRIm score would provide enhanced risk stratification, facilitating the early identification of children at greatest risk of delayed discharge, thereby informing potential clinical management pathways.

## Methods

### Study design and cohort participants

The data of the present retrospective cohort study were collected from 786 children and adolescents with MPP admitted to the Affiliated Hospital of Shandong University of Traditional Chinese Medicine during the period between 2014 and 2024. In these pediatric patients, one patient younger than 1 month or older than 18 years, 353 patients coinfected with other pathogens, four patients combined with chronic pulmonary infection, heart disease, coagulation disorders, autoimmune diseases/other immune diseases, or other immunodeficiency diseases, 32 patients missing measurement of lactate dehydrogenase levels, and five patients missing measurement of Alb levels were further excluded. Finally, 389 eligible pediatric patients were included for analysis. This study was approved by the Institutional Ethics Committee from the First College of Clinical Medicine, Shandong University of Traditional Chinese Medicine (approval no. 2020049). Written informed consent for participants was not required due to the retrospective nature of the study. All methods were performed in accordance with the relevant guidelines and regulations. Clinical trial number: not applicable.

### Study endpoints

The primary endpoint was the LOS, defined as the number of days from hospital admission to discharge. To comprehensively assess the association between exposure factors and the LOS, we employed a dual analytical approach. The LOS was analyzed as a continuous variable to quantify the absolute change in hospitalization days associated with each unit increase in the GRIm score, providing a precise measure of effect size. Concurrently, the LOS was dichotomized using two clinically relevant thresholds to evaluate its association with meaningful clinical outcomes: prolonged hospitalization was defined as an LOS ≥7 days, reflecting a guideline-recommended duration for pediatric pneumonia (18), and extended hospitalization as an LOS ≥11 days, corresponding to the 75th percentile in our cohort and capturing severe cases with complications and substantial resource utilization.

### Data collection and GRIm score calculation

We conducted data collection encompassing demographic information (age, gender, height, and weight), characteristics of diseases [onset seasons, duration of fever (onset of fever until admission), clinical symptoms (cough, wheeze, and hyperpyrexia), extrahepatic complications (liver function lesion, rash, and myocarditis), lung signs (pulmonary rales and diminished breath sounds)], vital signs (hypertension, respiratory rate, and body temperature), laboratory parameters (blood routine examination, hepatorenal function, and myocardial enzyme), and imaging manifestations (pulmonary atelectasis assessed by computed tomography, pulmonary infiltrate, and pleural effusion). We also collected the treatment information during admission, including the use of glucocorticoids, antibiotics, immunoglobulin, bronchoscopy, conventional oxygen therapy, and mechanical ventilation.

The exposure factor of the present was the GRIm score, which was structured following the methodology outlined by Bigot et al. (17). This index was based on three biomarkers: NLR, LDH levels, and serum Alb concentration. Among them, the NLR was the ratio of neutrophil count to lymphocyte count. Alb levels below the cutoff value (38.1 g/dl) were assigned 1 point; otherwise, it was 0 point. The levels of the NLR (1.44) and LDH (246 U/L) above their respective cutoff values were assigned 1 point, otherwise 0 point. Cutoffs for the NLR (1.44), LDH (246 U/L), and Alb (38.1 g/dl) were determined using maximally selected rank statistics within our cohort (https://cran.r-project.org/web/packages/maxstat/index.html), ensuring optimal discrimination for delayed discharge prediction in pediatric MPP. The total GRIm score was the sum of the scores for the NLR, LDH, and Alb (range 0–3 points), with higher scores indicating worse physical status.

### Statistical analysis

The normality of quantitative data was tested using skewness and kurtosis. Normally distributed quantitative data were described by mean and standard deviation (SD), while data with nonnormal distribution were described as medium and quartiles [M (Q1, Q3)]. Categorical variables were represented as number and percentage [*n* (%)].

To analyze the association between the GRIm score (and its components) with the LOS, we employed distinct statistical models tailored to the format of the outcome variable. For the analysis of the LOS as a continuous variable, we used multivariable linear regression models. The results were presented as *β* coefficients with 95% confidence intervals (CIs), representing the absolute change in the LOS (in days) associated with a one-unit increase in the exposure variable. For the analysis of the LOS as a binary outcome, defined as an LOS ≥7 and ≥11 days, we used multivariable logistic regression models. The results were presented as odds ratios (ORs) with 95% CIs.

The maximally selected test was utilized to select the best cutoff value of Alb, NLR, and LDH. This method was to test different cutoff values and select the cutoff value with the largest test statistic in statistical significance as the optimal cutoff value (19). Missing data were present for some variables in our dataset. To ensure data integrity and statistical power, we employed a stratified handling strategy based on the extent of missingness. Variables with a high missingness percentage (>20%), such as red cell distribution width (RDW), fibrinogen, prothrombin time, D-dimer, duration of fever, systolic/diastolic blood pressure, erythrocyte sedimentation rate (ESR), intermittent fever duration, and C-reactive protein (CRP), were excluded from the analysis altogether (i.e., variable deletion). For variables with a low to moderate missingness percentage (≤20%), missing data were imputed using the random forest method (via the missForest package in R). In total, missing values for 17 variables (including uric acid, globulin, creatinine, and monocyte count) were imputed. The complete list of all variables, their specific missing counts and percentages, and their respective handling methods (imputation or deletion) are detailed in Supplementary Table S1. To validate the reliability of the imputation, the distribution of each variable before and after imputation was compared; no significant differences were observed (all *P* > 0.05), indicating that the imputation process preserved the original characteristics of the dataset (see Supplementary Table S2 for sensitivity analysis).

Multivariable linear regression was performed to analyze the association between the GRIm score and the LOS, recognizing that LOS data can often be right-skewed. To assess the validity of this approach, we examined the distribution of the LOS variable and the distribution of the residuals from the final fitted linear model. Despite a right-skewed raw LOS distribution, the residuals of our final model approximated a normal distribution (as assessed by the Shapiro–Wilk test, *P* > 0.05), satisfying the key assumption of linear regression. We confirmed the linearity assumption by visually inspecting partial regression plots for the GRIm score against the LOS, which showed a reasonably linear relationship after accounting for covariates. Given that the model assumptions were reasonably met, and linear regression provides easily interpretable coefficients (*β*, representing the absolute change in days), we deemed it an appropriate and robust method for our primary analysis.

Covariates for adjustment in the multivariable linear regression models (for the continuous LOS outcome) were selected *a priori* based on their clinical relevance and established importance in previous literature on pneumonia outcomes. To provide a transparent overview of the unadjusted associations in our dataset, we first performed univariate linear regression analyses between all available baseline and treatment variables and the continuous LOS outcome. The results of these analyses are presented in Supplementary Table S3, which reports *β* coefficients and 95% confidence intervals. However, variable selection for the final multivariable model was not based on the statistical significance (*P*-value) observed in these univariate analyses. Instead, a prespecified set of covariates was chosen based on clinical grounds to avoid introducing selection bias. The selected covariates included the following: heart rate (a marker of physiological stress), preadmission glucocorticoid therapy and onset season (potential predictors of disease severity and presentation), imagological examination findings (a direct indicator of pulmonary involvement), and key in-hospital treatments including postadmission glucocorticoid therapy, duration of macrolide therapy, use of other antibiotics, and use of immunoglobulin (which directly influence the course of hospitalization and are strong potential confounders).

Furthermore, we evaluated the predictive performance of the GRIm score against its individual components (Alb, NLR, and LDH) for the binary outcomes (LOS ≥7 and ≥11 days) using ROC curves, NRI, and IDI. The NRI was used to quantify the improvement in risk stratification accuracy when using the GRIm score compared with single markers. IDI measured the increased separation between outcome groups. An NRI >0 and IDI >0 indicate a significantly improved predictive power of the GRIm score.

All statistical analyses were performed using SAS 9.4 (SAS Institute Inc., Cary, NC, USA) and R version 4.2.2 (Institute for Statistics and Mathematics, Vienna, Austria). Statistical differences were considered when the *P*-value was <0.05.

## Results

### Baseline characteristics of children and adolescents with MPP

**Figure 1:**
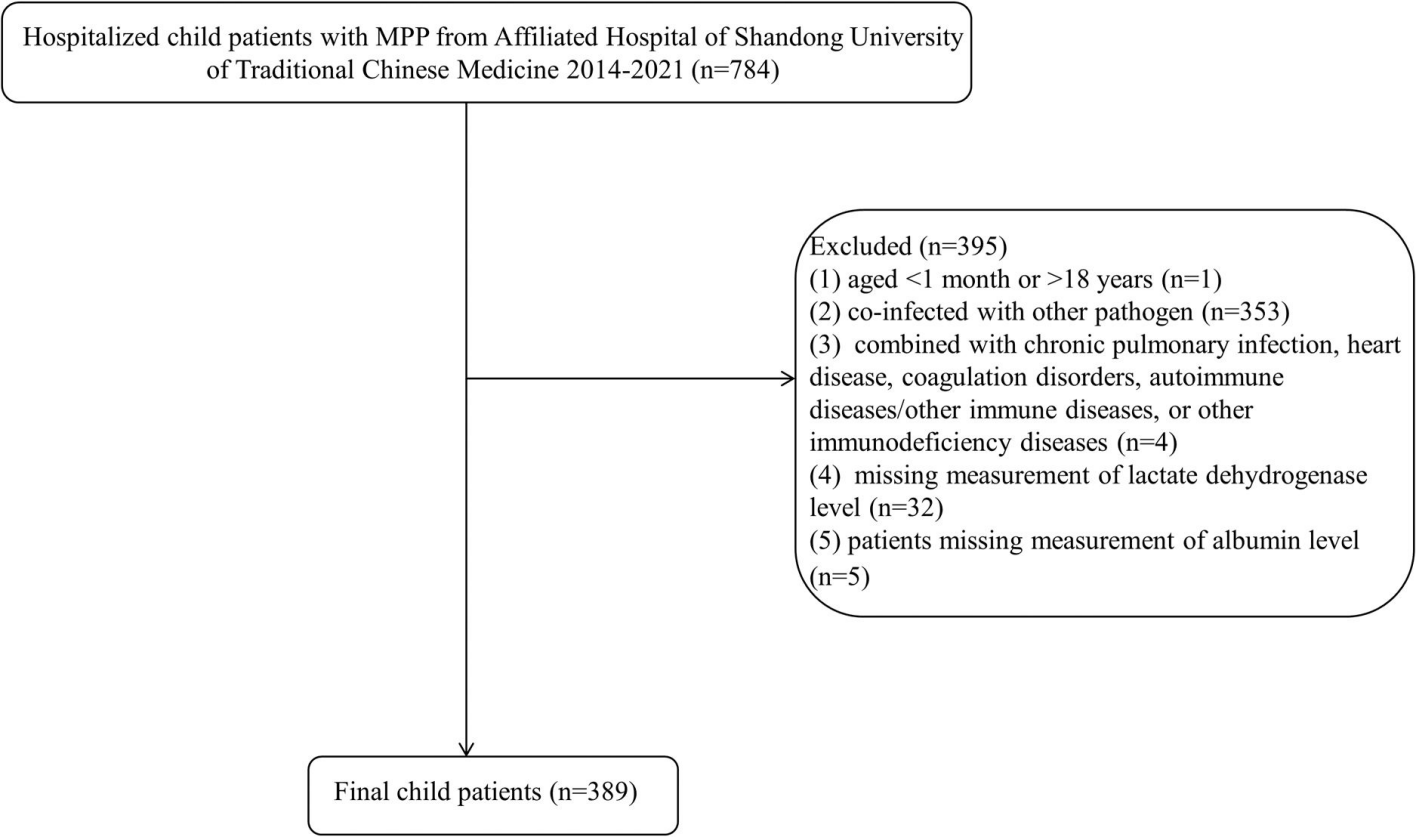
Flowchart of patient selection and inclusion process for the study cohort.

A flowchart of study children and adolescents with MPP is exhibited in Figure 1. A total of 389 eligible pediatric patients with MPP were included in the final analysis (Table 1). The cohort had a mean age of 5.21 ± 2.59 years with balanced gender distribution (51.16% male). Critically, biomarkers comprising the GRIm score demonstrated significant abnormalities: 63.24% of patients exhibited an elevated NLR (≥1.44), 39.85% had increased LDH (≥246 U.L), and 14.65% showed hypoalbuminemia (<38.1 g/dl).

**Table 1:** Characteristics of study children and adolescents with MPP.

Variables	Total (*N* = 389)
Age, years, mean (±SD)	5.21 (±2.59)
Weight, kg, mean (±SD)	22.11 (±9.93)
Respiratory rate, bpm, M (Q₁, Q₃)	23.00 (21.00, 26.00)
Temperature, °C, mean (±SD)	37.22 (±0.93)
Heart rate, bpm, mean (±SD)	111.76 (±15.00)
Hemoglobin, g/L, mean (±SD)	128.50 (±10.53)
RBC, 10^12^/L, mean (±SD)	4.62 (±0.38)
WBC, 10^12^/L, mean (±SD)	8.16 (±3.39)
Platelet, 10^12^/L, mean (±SD)	279.60 (±81.19)
Monocyte, 10^12^/L, *M* (Q₁, Q₃)	0.64 (0.46, 0.83)
GGT, U/L, mean (±SD)	12.54 (±2.82)
ALP, U/L, mean (±SD)	190.12 (±57.90)
TBIL, µmol/L, *M* (Q₁, Q₃)	7.70 (6.20, 9.60)
TP, g/L, mean (±SD)	66.52 (±4.92)
Globulin, g/L, mean (±SD)	25.65 (±3.72)
Creatinine, µmol/L, mean (±SD)	29.77 (±7.82)
Uric acid, µmol/L, mean (±SD)	267.92 (±70.13)
HS-CRP, mg/L, *M* (Q₁, Q₃)	4.90 (1.50, 10.40)
Alpha HBDH, U/L, mean (±SD)	171.06 (±38.33)
Gender, *n* (%)	
Female	190 (48.84)
Male	199 (51.16)
Preglucocorticoid therapy, *n* (%)	
No	336 (86.38)
Yes	53 (13.62)
Premacrolides, *n* (%)	
No	241 (61.95)
Yes	148 (38.05)
Prebeta lactam, *n* (%)	
No	288 (74.04)
Yes	101 (25.96)
Onset season, *n* (%)	
Autumn	96 (24.68)
Spring	72 (18.51)
Summer	62 (15.94)
Winter	159 (40.87)
Precough, *n* (%)	
No	8 (2.06)
Yes	381 (97.94)
Prewheezing, *n* (%)	
No	357 (91.77)
Yes	32 (8.23)
Prehigh fever, *n* (%)	
No	260 (66.84)
Yes	129 (33.16)
Extrapulmonary complication, *n* (%)	
No	341 (87.66)
Yes	48 (12.34)
Pulmonary signs, *n* (%)	
Others	115 (29.56)
Pulmonary rales	274 (70.44)
Imagological examination, *n* (%)	
Bilateral	93 (23.91)
No/unknown	244 (62.72)
Unilateral	52 (13.37)
Postglucocorticoid therapy, *n* (%)	
No	61 (15.68)
Yes	328 (84.32)
Postmacrolides length, days, mean (±SD)	6.00 (±2.82)
Post other antibiotics, *n* (%)	
No	297 (76.35)
Yes	92 (23.65)
Postimmune globulin, *n* (%)	
No	360 (92.54)
Yes	29 (7.46)
Postconventional oxygen therapy, *n* (%)	
No	367 (94.34)
Yes	22 (5.66)
AST/ALT, mean (±SD)	2.49 (±0.97)
Alb, g/L, mean (±SD)	40.83 (±2.83)
Alb, *n* (%)	
<38.1	57 (14.65)
≥38.1	332 (85.35)
NLR, *M* (Q₁, Q₃)	1.89 (1.06, 3.01)
NLR, *n* (%)	
<1.4444	143 (36.76)
≥1.4444	246 (63.24)
LDH, U/L, mean (±SD)	240.18 (±55.10)
LDH, *n* (%)	
<246	234 (60.15)
≥246	155 (39.85)
GRIm score, score, mean (±SD)	1.18 (±0.76)
LOS, days, mean (±SD)	7.03 (±2.74)
LOS, days, *n* (%)	
<11	339 (87.15)
≥11	50 (12.85)
LOS, days, *n* (%)	
<7	187 (48.07)
≥7	202 (51.93)

SD, standard deviation; M, median; Q1, 1st Quartile; Q3, 3st Quartile; MPP, mycoplasma pneumoniae pneumonia; RBC, red blood cell; WBC, white blood cell; GGT, gamma-glutamyl transpeptidase; ALP, alkaline phosphatase; TBIL, total bilirubin; TP, total protein; HS-CRP, high-sensitivity C-reactive protein; AST/ALT, aspartate aminotransferase/alanine aminotransferase; Alb, albumin; NLR, neutrophil/lymphocyte ratio; LDH, lactate dehydrogenase; GRIm score, Gustave Roussy Immune Score; LOS, length of stay.

The cohort exhibited substantial disease severity, evidenced by high rates of systemic inflammation (median HS-CRP 4.90 mg/L) and extrapulmonary complications (12.34%). Treatment intensity was notable: 84.32% received glucocorticoids and 23.65% required additional antibiotics, while 5.66% needed oxygen support.

Importantly, over half (51.93%) experienced prolonged hospitalization (LOS ≥7 days), and 12.85% had extended stays ≥11 days, confirming the clinical relevance of delayed discharge prediction in this population.

### Association of the GRIm score and components with the LOS among children and adolescents with MPP

**Figure 2:**
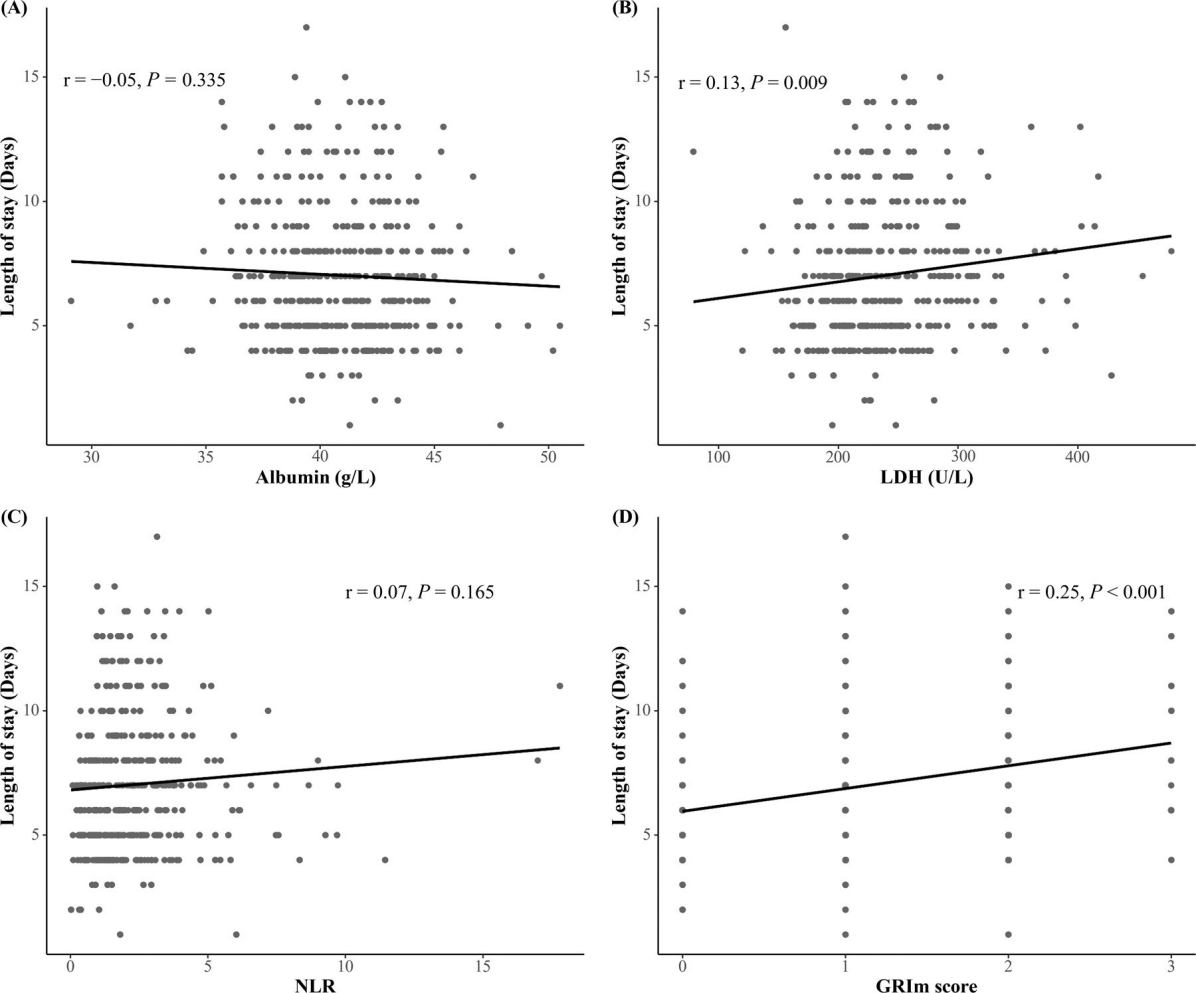
**(A–D)** Scatter plots showing the unadjusted correlation between the GRIm score and its component with the LOS. The solid line represents the line of best fit from a linear model, and the shaded area represents the 95% CI. While the raw LOS data exhibit some right skewing, the positive correlation between the GRIm score and the LOS is evident. The relationship appears broadly linear, supporting its use in linear regression models.

The associations between the GRIm score (and its individual components) and the LOS were analyzed using statistical models appropriate for the format of the outcome. Multivariable linear regression analysis, adjusted for clinical confounders, indicated that elevated LDH (≥246 U/L) was significantly associated with a prolonged LOS (*β* = 0.40, 95%CI: 0.00–0.80, *P* = 0.049), whereas neither Alb nor the NLR demonstrated significant independent associations (all *P* > 0.05). Importantly, the composite GRIm score exhibited a robust dose-dependent relationship with a 0.33-day extension in the LOS (*β* = 0.33, 95% CI: 0.07–0.58, *P* = 0.013); this effect persisted after adjustment for treatment variations (Table 2). In parallel analyses of dichotomized outcomes, multivariable logistic regression models confirmed that the GRIm score was significantly associated with increased odds of both prolonged (LOS ≥7 days) and extended (LOS ≥11 days) hospitalization, as detailed in Table 3. The positive association between the GRIm score and the continuous LOS was further supported visually by a scatter plot, which demonstrated a stronger correlation compared with LDH alone (Figures 2A–D), underscoring the advantage of the integrated score in capturing the variance in hospitalization burden.

**Table 2:** Association between LDH, Alb, NLR, and GRIm score with the LOS among children and adolescents with MPP.

Variables	Model 1		Model 2	
	*β* (95% CI)	*P*	*β* (95% CI)	*P*
LDH	0.01 (0.00–0.01)	0.009	0.00 (−0.00–0.01)	0.255
LDH				
<246	Ref		Ref	
≥246	0.95 (0.40–1.50)	0.001	0.40 (0.00–0.80)	0.049
Alb	−0.05 (−0.14–0.05)	0.335	−0.03 (−0.09–0.04)	0.416
Alb				
<38.1	Ref		Ref	
≥38.1	−0.37 (−1.14–0.40)	0.345	−0.12 (−0.65–0.41)	0.660
NLR	0.10 (−0.04–0.23)	0.165	0.01 (−0.08–0.11)	0.816
NLR				
<1.4444	Ref		Ref	
≥1.4444	1.09 (0.54–1.65)	<0.001	0.31 (−0.09–0.72)	0.126
GRIm-score	0.92 (0.57–1.26)	<0.001	0.33 (0.07–0.58)	0.013

MPP, mycoplasma pneumoniae pneumonia; LDH, lactate dehydrogenase; NLR, neutrophil/lymphocyte ratio; Alb, albumin; GRIm score, Gustave Roussy Immune Score; LOS, length of stay; Ref, reference; CI, confidence interval.

Model 1, crude model;

Model 2, adjusted heart rate, preglucocorticoid therapy, onset season, imagological examination, postglucocorticoid therapy, postmacrolides length, post other antibiotics and postimmune globulin.

**Table 3:** Multivariable-adjusted associations of the GRIm score and its components with prolonged (LOS ≥7 and ≥11 days).

Outcome	Variable	Adjusted OR (95% CI)	*P*	AUC (95% CI)
LOS ≥7 days	Alb	1.00 (0.93–1.07)	0.894	0.492 (0.434–0.549)
	LDH	1.01 (1.00–1.01)	0.002	0.604 (0.548–0.660)
	NLR	1.13 (1.01–1.27)	0.027	0.602 (0.545–0.658)
	GRIm score	1.98 (1.49–2.64)	<0.001	0.635 (0.585–0.685)
LOS ≥11 days	Alb	0.95 (0.86–1.06)	0.365	0.541 (0.457–0.625)
	LDH	1.00 (1.00–1.01)	0.223	0.578 (0.499–0.657)
	NLR	1.07 (0.94–1.21)	0.319	0.580 (0.511–0.650)
	GRIm score	1.81 (1.22–2.68)	0.003	0.622 (0.543–0.700)

GRIm score, Gustave Roussy Immune Score; Alb, albumin; LOS, length of stay; LDH, lactate dehydrogenase; NLR, neutrophil/lymphocyte ratio; Ref, reference; CI, confidence interval; AUC, area under the curve.

All presented ORs and AUC values are derived from multivariable-adjusted models.

Continuous variables were dichotomized using the following cutoff values: Albumin (<38.1 vs. ≥38.1 g/L), LDH (<246 vs. ≥246 U/L), Neutrophil-to-lymphocyte ratio, NLR (<1.44 vs. ≥1.44). The Gustave Roussy Immune Score (GRIm score) is an ordinal variable ranging from 0 to 3.

Model adjusted for heart rate, preglucocorticoid therapy, onset season, imagological examination, postglucocorticoid therapy, postmacrolides length, post other antibiotics and postimmune globulin.

### Comparing the association strength between the GRIm score and its components with the LOS in children with MPP

The composite GRIm score demonstrated a significantly stronger association with prolonged hospitalization than its individual components. For both clinical thresholds (LOS ≥7 and ≥11 days), each point increase in the GRIm score was associated with a substantially elevated odds of delayed discharge (OR = 1.98, 95% CI: 1.49–2.64, *P* < 0.001 and OR = 1.81, 95% CI: 1.22–2.68, *P* = 0.003, respectively) (Table 3). This association was more consistent and robust than those observed for any single biomarker, which was either weak or non-significant.

**Figure 3:**
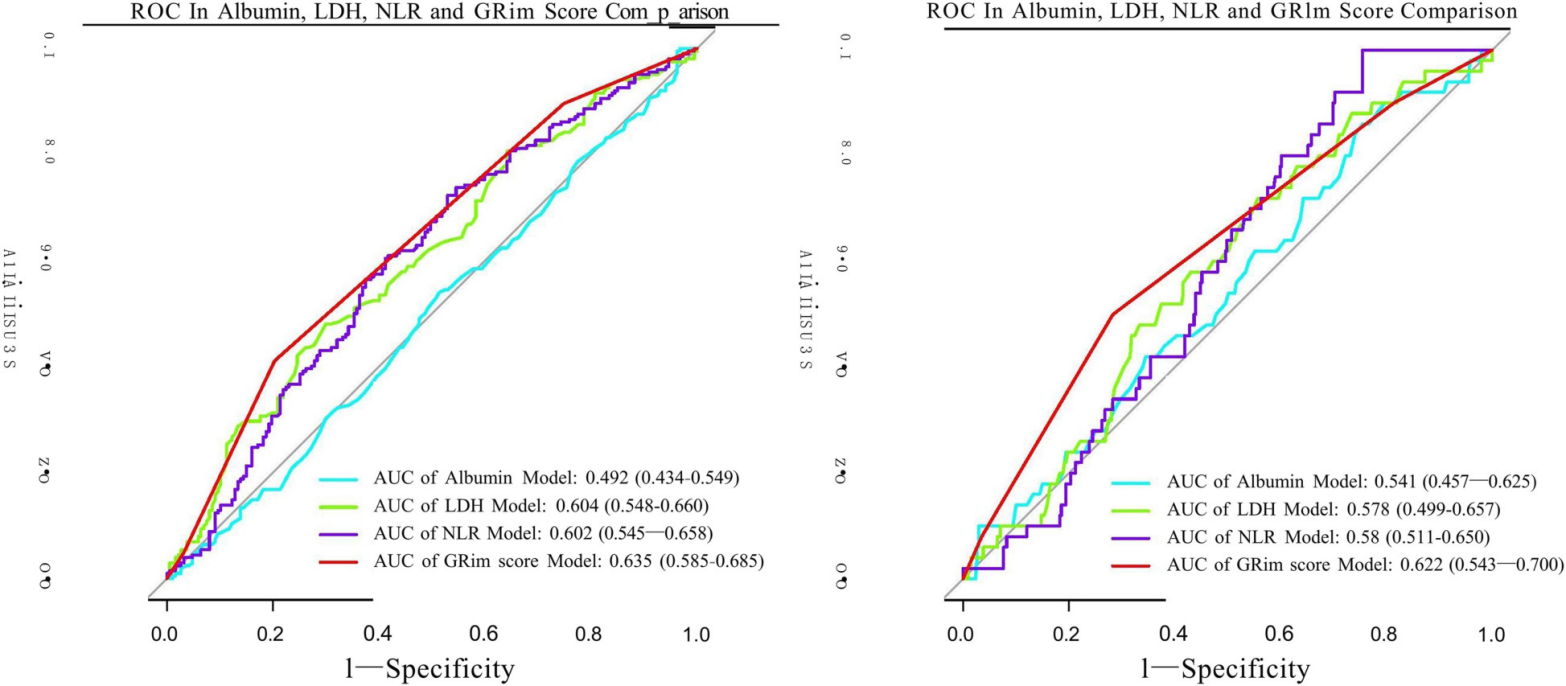
ROC curves assessing the discriminative ability of the GRIm score and its individual components for predicting prolonged hospitalization. **(a)** ROC curves for the outcome of LOS ≥7 days. (**b)** ROC curves for the outcome of LOS ≥11 days. GRIm score, Gustave Roussy Immune Score; LOS, length of stay; MPP, mycoplasma pneumoniae pneumonia; CI, confidence interval; ROC, receiver operating characteristic; AUC, area under the curve.

Consistent with these findings, the ability of the GRIm score to discriminate between patients, while poor in absolute terms (AUC = 0.635 for LOS ≥7 days; AUC = 0.622 for LOS ≥11 days), was statistically superior to that of any single component (NLR, LDH, or Alb) (Figure 3). This relative advantage was further quantified by reclassification metrics. The integrated GRIm score provided significantly better risk stratification compared with isolated biomarkers, as evidenced by significantly negative NRI and IDI values (all *P* < 0.05) when each component was compared with the full score (Table 4). Specifically, the GRIm score yielded absolute improvements in risk classification of 3.39%–6.08% for an LOS ≥7 days and 2.07%–2.24% for an LOS ≥11 days.

**Table 4:** Comparison of the predictive power of the GRIm score and components for an LOS ≥7 days and an LOS ≥11 days.

Outcome	Mode 1	NRI (95% CI)	*P*	IDI (95% CI)	*P*
LOS ≥7 days	Alb	−0.1474 (−0.2225, −0.0723)	<0.001	−0.0608 (−0.0843, −0.0373)	<0.001
	LDH	−0.14 (−0.2183, −0.0618)	<0.001	−0.0339 (−0.0562, −0.0117)	0.003
	NLR	−0.2136 (−0.3195, −0.1078)	<0.001	−0.047 (−0.0707, −0.0232)	<0.001
	GRIm score	Ref		Ref	
LOS ≥11 days	Alb	−0.1876 (−0.3311, −0.0441)	0.010	−0.0224 (−0.0379, −0.0069)	0.005
	LDH	−0.2289 (−0.3851, −0.0727)	0.004	−0.0207 (−0.0358, −0.0056)	0.007
	NLR	−0.2735 (−0.4233, −0.1237)	<0.001	−0.0217 (−0.0393, −0.0042)	0.015
	GRIm score	Ref		Ref	

GRIm score, Gustave Roussy Immune Score; Alb, albumin; LOS, length of stay; LDH, lactate dehydrogenase; NLR, neutrophil/lymphocyte ratio; Ref, reference; CI, confidence interval; NRI, net reclassification improvement; IDI, integrated discrimination improvement.

Continuous variables were dichotomized using the following cutoff values: Albumin (<38.1 vs. ≥38.1 g/L), LDH (<246 vs. ≥246 U/L), neutrophil-to-lymphocyte ratio, NLR (<1.44 vs. ≥1.44). The Gustave Roussy Immune Score (GRIm score) is an ordinal variable ranging from 0 to 3.

Together, these results establish that integrating inflammatory and nutritional markers into the GRIm score provides a more powerful measure of association with a prolonged LOS and enhances risk stratification compared with using any component alone, despite its limited overall discriminative capacity.

## Discussion

This retrospective cohort study enrolled 389 children and adolescents with MPP admitted to the Affiliated Hospital of Shandong University of Traditional Chinese Medicine between 2014 and 2021. Our findings demonstrate that the GRIm score, a composite biomarker integrating the NLR and LDH and Alb levels, is significantly associated with prolonged hospitalization in these patients. Importantly, our analysis shows a stronger association with the LOS compared with any its individual components, as evidenced by its superior discriminative performance (AUC: 0.635 vs. ≤0.604 for LOS ≥7 days) and improving risk reclassification by 3.39%–6.08% (NRI/IDI *P* < 0.05). These results suggest that while individual biomarkers such as the NLR and LDH reflect isolated aspects of MPP severity, the GRIm score may better capture the integrated immune-nutritional dysfunction contributing to delayed recovery.

In China, the incidence of MPP accounts for about 10%–40% of CAP in children. It is worth noting that MPP can be a regional or even a nationwide phenomenon, with a cycle of about every 3–7 years (20, 21). Although MPP is usually considered a self-limiting disease, due to the increasingly serious phenomenon of antibiotic resistance, some children diagnosed with MPP may continue to be infected with MP for months or years, and at the same time experience airway hyperresponsiveness and even develop asthma, which greatly increases medical and economic costs (22, 23). Therefore, in order to reasonably control the LOS of patients with MPP, optimize hospital efficiency, and improve treatment outcomes, it is of great clinical significance to explore simple economic indicators related to it. A retrospective cohort study in 2017 proposed a GRIm score based on the combined use of Alb, NLR, and LDH in order to better select patients who benefited from the phase I trial of immune checkpoint therapy (17). The results showed that patients with a higher GRIm score had a significantly shorter overall survival (OS) rate, and in a prospective validation study of 113 patients, patients with a high GRIm score again showed a poor OS rate. In current clinical studies, the GRIm score is mostly used to predict the prognosis of cancer patients, including gastric cancer patients (24), small cell lung cancer patients receiving immunotherapy (25), and esophageal squamous cell carcinoma patients (26). However, as far as we know, there are no studies on the predictive value of the GRIm score for determining the LOS in children and adolescents with MPP. In the present study, we retrospectively analyzed the association between the GRIm score and the LOS in patients with MPP and found that the GRIm score was significantly associated with the LOS of such patients. Moreover, we also found that the GRIm score, instead of the NLR, LDH and Alb, was a useful indicator to predict the LOS of children with MPP.

Although the pathogenic mechanisms of MPP have not been fully elucidated, cytotoxicity, adhesion damage, and inflammatory damage are important pathogenic mechanisms that lead to the occurrence and development of MPP (4). The NLR, LDH, and Alb are routine clinical test indicators. Neutrophils can activate acute inflammatory responses by phagocytosis, release of cytokines, and extracellular capture. Moreover, neutrophils can obtain relevant information from the body and macrophages, participate in the activation and regulation of immune cells, and therefore play an important role in infection, autoimmunity, chronic inflammation, and tumors (27). A large multicenter cohort study compared the prognostic predictive ability of 16 inflammatory/nutritional indicators in lung cancer patients and indicated that the NLR had great ability in predicting the overall survival rates of patients (28). Nutrition is also a factor that is easily overlooked in children with MPP. Alb plays a role in nutrition, transportation, maintaining plasma pH, and immunity, and its concentration changes are related to its response to tissue damage, inflammation, infection, and various malignancies (13). Hypoalbuminemia can lead to nutrient transport disorders. At this time, Alb not only reflects malnutrition or excessive catabolism, but also reflects the degree of systemic inflammation. LDH exists as a cytoplasmic enzyme in various important organs. When cells are dissolved or cell membranes are damaged, LDH can be released outside the cells, causing an increase in serum LDH, which may be one of the predictive indicators of the severity of inflammation. Some scholars have used LDH as a prognostic marker for diseases related to mycoplasma pneumoniae infection, especially refractory MPP (29, 30).

The GRIm score integrates the NLR, Alb, and LDH—parameters reflecting inflammatory intensity, nutritional status, and tissue injury, all implicated in the immune and metabolic response to infection. Our analysis reveals that this composite score exhibits a consistent and independent association with the LOS in pediatric patients with MPP, demonstrating a stronger association than any of its individual components. Although LDH alone shows limited association at certain thresholds, and neither the NLR nor Alb alone reaches statistical significance, the integrated GRIm score is significantly associated with an increased LOS (*β* = 0.33, *P* = 0.013). Furthermore, while the overall discriminative ability of the score remains limited (AUC: 0.62–0.63), it shows statistically significant—although modest—improvements in reclassification compared with single biomarkers (NRI/IDI between 3.4% and 6.1%, *P* < 0.05). These findings suggest that the GRIm score may hold potential as a risk-stratification tool to help identify children at higher risk of extended hospitalization. For example, patients with elevated scores could potentially be considered for closer monitoring (e.g., vital signs every 4–6 h and daily assessment of CRP, LDH, and albumin) and might benefit from nutritional support such as albumin supplementation if levels are low. Those with lower scores may be suitable for standardized management with the possibility of early discharge. If validated, such an approach could assist in optimizing inpatient resource allocation, particularly during outbreaks.

Our study has several limitations that should be acknowledged. First, the single-center retrospective design inherently introduces risks of selection bias and unmeasured confounding, despite our efforts to adjust for known clinical variables. The generalizability of our findings requires validation in multicenter, prospective cohorts. Second, while BMI was unavailable retrospectively, albumin—as an acute-phase reactant and nutritional marker—more directly captures infection-induced catabolism relevant to the LOS than anthropometric indices (31). Third, more critically, we must address several methodological limitations pertaining to our statistical approach. Linear regression: although we utilized multivariable linear regression to determine the continuous LOS outcome because of its straightforward clinical interpretability, we recognize that its assumptions may not be perfectly suited for our data. The weak, and potentially non-linear, bivariate correlations (Figures 2A–D) suggest that our model might not fully capture the complex relationships between inflammatory markers and the LOS. While the model residuals met normality assumptions, future studies with larger samples could employ more flexible techniques to better model the right-skewed distribution of the LOS and uncover any non-linear effect. The use of ORs: For our binary outcome of delayed discharge (LOS ≥7 days), we reported ORs derived from logistic regression. Given the high prevalence of this outcome (51.9%) in our cohort, it is well-established that the OR can substantially overestimate the relative risk (RR), potentially misleading the interpretation of the effect size. We opted for logistic regression for its computational stability during model fitting with multiple covariates. However, we explicitly acknowledge that the reported ORs should be interpreted as measures of association strength rather than direct estimates of risk increase. The findings would be more accurately quantified using methods like modified Poisson regression to report RRs, and we advise caution in interpreting the magnitude of these effect estimates. With regard to model discrimination, the discriminative ability of the GRIm score, with an AUC of 0.62–0.63, must be classified as “poor” according to standard criteria, indicating that it is unsuitable as a stand-alone predictive tool at present. Nevertheless, the consistent and statistically significant association we identified across multiple analytical frameworks underscores that the GRIm score captures a meaningful biologic signal relevant to the clinical course of MPP. Furthermore, the GRIm score itself does not elucidate any underlying immune-inflammatory mechanisms and may be influenced by comorbidities and other unmeasured factors. Future studies incorporating detailed immune profiling, comorbidity indices, and prospective multicenter designs are needed to refine and validate the utility of the score in predicting the LOS and other clinical outcomes in pediatric MPP.

## Conclusion

Our study demonstrates that the GRIm score at admission is independently associated with delayed discharge in children and adolescents with MPP, showing a stronger association than any of its individual components. This suggests that the integration of inflammatory and nutritional markers into a single composite score provides a more robust measure of disease severity related to hospitalization length. However, the overall discriminative ability of the score currently does not support its use as a stand-alone predictive tool. The findings from this retrospective cohort require validation in large-scale, multicenter prospective studies before any potential clinical application can be considered.

## Data Availability

The raw data supporting the conclusions of this article will be made available by the authors without undue reservation.
