# Supplementary material for: Association between Gustave Roussy Immune Score and delay in discharge among children and adolescents with mycoplasma pneumoniae pneumonia: a retrospective cohort study
Source: Front Pediatr. 2025 Oct 3;13:1564217. doi: 10.3389/fped.2025.1564217 (PMC12531050; doi:10.3389/fped.2025.1564217)
Supplement: Supplementary file 1 [file Supplementaryfile1.docx]

Table S1 Description of missing variables

| Variables | Total sample of missing variable | Percentage of missing variable | Handle methods |
| --- | --- | --- | --- |
| Uric acid | 1 | 0.26% | random forest method |
| Globulin | 1 | 0.26% | “ |
| Creatinine | 1 | 0.26% | “ |
| GGT | 2 | 0.51% | “ |
| ALP | 2 | 0.51% | “ |
| TBIL | 2 | 0.51% | “ |
| Alpha HBDH | 2 | 0.51% | “ |
| WBC | 3 | 0.77% | “ |
| TP | 3 | 0.77% | “ |
| AST | 3 | 0.77% | “ |
| Platelet | 18 | 4.63% | “ |
| Hemoglobin | 25 | 6.43% | “ |
| RBC | 31 | 7.97% | “ |
| HS-CRP | 70 | 17.99% | “ |
| Monocyte | 75 | 19.28% | “ |
| Eosinophil | 95 | 24.42% | eliminate |
| RDW | 101 | 25.96% | “ |
| Fibrinogen | 115 | 29.56% | “ |
| APTT | 115 | 29.56% | “ |
| PT | 115 | 29.56% | “ |
| D-Dimer | 117 | 30.08% | “ |
| Heating duration | 144 | 37.02% | “ |
| DBP | 165 | 42.42% | “ |
| SBP | 165 | 42.42% | “ |
| ESR | 273 | 70.18% | “ |
| Intermittent heating time | 280 | 71.98% | “ |
| Height | 317 | 81.49% | “ |
| CRP | 333 | 85.60% | “ |

Note,

“= ditto mark, indicates the same value as the cell above.

GGT, gamma-glutamyl transpeptidase; ALP, alkaline phosphatase; TBIL, total bilirubin; HBDH, hydroxybutyrate dehydrogenase; WBC, white blood cell; TP, total potential; AST, aspartate aminotransferase; RBC, red blood cell; HS-CRP, high-sensitivity C-reactive protein; RDW, red cell distribution width; APTT, activated partial thromboplastin time; PT, prothrombin time; DBP, diastolic blood pressure; SBP, systolic blood pressure; ESR, erythrocyte sedimentation rate; CRP, C-reactive protein.

Table S2 Sensitivity analyses before and after imputation of missing data

| Variables | Before imputation | After imputation | Statistics | *P* |
| --- | --- | --- | --- | --- |
| Hemoglobin, Mean (±SD) | 128.32 (±10.73) | 128.50 (±10.53) | t = 0.223 | 0.823 |
| Monocyte, M (Q₁, Q₃) | 0.60 (0.44, 0.82) | 0.64 (0.46, 0.83) | W = 64276.500 | 0.231 |
| Creatinine, Mean (±SD) | 29.77 (±7.83) | 29.77 (±7.82) | t = 0.003 | 0.997 |
| Globulin, Mean (±SD) | 25.64 (±3.72) | 25.65 (±3.72) | t = 0.016 | 0.988 |
| TP, Mean (±SD) | 66.54 (±4.93) | 66.52 (±4.92) | t = -0.061 | 0.951 |
| ALP, Mean (±SD) | 190.06 (±58.04) | 190.12 (±57.90) | t = 0.015 | 0.988 |
| RBC, Mean (±SD) | 4.61 (±0.39) | 4.62 (±0.38) | t = 0.361 | 0.718 |
| Platelet, Mean (±SD) | 278.96 (±82.83) | 279.60 (±81.19) | t = 0.108 | 0.914 |
| TBIL, M (Q₁, Q₃) | 7.70 (6.20, 9.60) | 7.70 (6.20, 9.60) | W = 75134.000 | 0.965 |
| HS-CRP, M (Q₁, Q₃) | 3.50 (1.05, 9.35) | 4.90 (1.50, 10.40) | W = 66295.500 | 0.116 |
| WBC, Mean (±SD) | 8.17 (±3.41) | 8.16 (±3.39) | t = -0.015 | 0.988 |
| GGT, Mean (±SD) | 12.54 (±2.82) | 12.54 (±2.82) | t = -0.023 | 0.982 |
| Uric acid, Mean (±SD) | 267.94 (±70.21) | 267.92 (±70.13) | t = -0.004 | 0.997 |
| Alpha HBDH, Mean (±SD) | 171.00 (±38.41) | 171.06 (±38.33) | t = 0.022 | 0.982 |
| AST, M (Q₁, Q₃) | 30.00 (25.00, 36.00) | 30.00 (25.00, 36.00) | W = 75165.500 | 0.977 |

TP, total potential; ALP, alkaline phosphatase; RBC, red blood cell; TBIL, total bilirubin; HS-CRP, high-sensitivity C-reactive protein; WBC, white blood cell; GGT, gamma-glutamyl transpeptidase; HBDH, hydroxybutyrate dehydrogenase; AST, aspartate aminotransferase.

Table S3 Results of univariate linear regression analysis for the association between candidate variables and LOS (continuous outcome).

| Variables | Model 1 |  | Model 2 |  |
| --- | --- | --- | --- | --- |
|  | β (95% CI) | *P* | β (95% CI) | *P* |
| Weight | -0.002 (-0.03-0.02) | 0.835 |  |  |
| Respiratory rate | 0.005 (-0.03-0.04) | 0.743 |  |  |
| Temperature | 0.330 (0.04-0.62) | 0.028 |  |  |
| Heart rate^*^ | 0.020 (0.00-0.04) | 0.028 | 0.015 (0.00-0.03) | 0.015 |
| Hemoglobin | 0.010 (-0.02-0.04) | 0.491 |  |  |
| RBC | 0.840 (0.12-1.56) | 0.022 |  |  |
| WBC | 0.010 (-0.07-0.09) | 0.815 |  |  |
| Platelet | 0.000 (-0.00-0.00) | 0.907 |  |  |
| Monocyte | 0.651 (-0.00-1.31) | 0.051 |  |  |
| GGT | 0.050 (-0.05-0.15) | 0.305 |  |  |
| ALP | -0.005 (-0.01-0.00) | 0.364 |  |  |
| TBIL | -0.090 (-0.18--0.00) | 0.047 |  |  |
| TP | 0.015 (-0.04-0.07) | 0.592 |  |  |
| Globulin | 0.060 (-0.01-0.13) | 0.115 |  |  |
| Creatinine | -0.013 (-0.05-0.02) | 0.502 |  |  |
| Uric acid | -0.003 (-0.01--0.00) | 0.015 |  |  |
| HS-CRP | 0.025 (-0.00-0.05) | 0.095 |  |  |
| Alpha HBDH | 0.010 (0.00-0.02) | 0.003 |  |  |
| Gender |  |  |  |  |
| Female | Ref |  |  |  |
| Male | 0.220 (-0.33-0.77) | 0.430 |  |  |
| Age | 0.010 (-0.10-0.12) | 0.859 |  |  |
| Pre glucocorticoid therapy^*^ |  |  |  |  |
| No | Ref |  | Ref |  |
| Yes | 1.445 (0.66-2.23) | <0.001 | 0.610 (0.05-1.17) | 0.032 |
| Pre macrolides |  |  |  |  |
| No | Ref |  |  |  |
| Yes | -0.105 (-0.67-0.46) | 0.705 |  |  |
| Pre beta lactam |  |  |  |  |
| No | Ref |  |  |  |
| Yes | 0.315 (-0.31-0.94) | 0.320 |  |  |
| Onset season^*^ |  |  |  |  |
| Autumn | Ref |  | Ref |  |
| Spring | -1.130 (-1.96--0.30) | 0.008 | -0.490 (-1.06-0.08) | 0.092 |
| Summer | -1.300 (-2.17--0.43) | 0.004 | -0.495 (-1.09-0.10) | 0.104 |
| Winter | -0.810 (-1.50--0.12) | 0.021 | -0.700 (-1.17--0.23) | 0.004 |
| Pre cough |  |  |  |  |
| No | Ref |  |  |  |
| Yes | 1.185 (-0.74-3.11) | 0.228 |  |  |
| Pre wheezing |  |  |  |  |
| No | Ref |  |  |  |
| Yes | -0.035 (-1.03-0.96) | 0.943 |  |  |
| Pre high fever |  |  |  |  |
| No | Ref |  |  |  |
| Yes | 0.590 (0.01-1.17) | 0.046 |  |  |
| Extrapulmonary complication |  |  |  |  |
| No | Ref |  |  |  |
| Yes | 0.410 (-0.42-1.24) | 0.329 |  |  |
| Pulmonary signs |  |  |  |  |
| Others | Ref |  |  |  |
| Pulmonary rales | 0.565 (-0.03-1.16) | 0.063 |  |  |
| Imageological examination^*^ |  |  |  |  |
| Bilateral | Ref |  | Ref |  |
| No/Unknown | -1.310 (-1.94--0.68) | <0.001 | -0.885 (-1.34--0.43) | <0.001 |
| Unilateral | 0.785 (-0.11-1.68) | 0.085 | 0.525 (-0.11-1.16) | 0.104 |
| Post glucocorticoid therapy^*^ |  |  |  |  |
| No | Ref |  | Ref |  |
| Yes | 2.005 (1.28-2.73) | <0.001 | 0.500 (-0.03-1.03) | 0.066 |
| Post macrolides length^*^ | 0.623 (0.55-0.70) | <0.001 | 0.580 (0.51-0.65) | <0.001 |
| Post other antibiotic^*^ |  |  |  |  |
| No | Ref |  | Ref |  |
| Yes | 1.710 (1.09-2.33) | <0.001 | 1.273 (0.82-1.73) | <0.001 |
| Post immune globulin^*^ |  |  |  |  |
| No | Ref |  | Ref |  |
| Yes | 1.680 (0.65-2.71) | 0.001 | 0.890 (0.18-1.60) | 0.014 |
| Post conventional oxygen therapy |  |  |  |  |
| No | Ref |  |  |  |
| Yes | 1.170 (-0.01-2.35) | 0.052 |  |  |
| AST/ALT | 0.130 (-0.15-0.41) | 0.369 |  |  |

Note,

this table presents the unadjusted associations between each variable and length of stay (in days) using univariate linear regression.

Model 1 represents the univariate linear regression model including all variables observations for each variable;

Model 2 presents the results for variables that were ultimately included in the final multivariable model (indicated by *), as derived from the same univariate regression approach for consistency and comparability. It is included here to show the unadjusted effect size of these selected variables prior to multivariable adjustment.

Variables were not selected for the final multivariable model based on the *P*-values presented here. The final covariates were chosen based on clinical rationale and are indicated in the table. All β coefficients have been reported to three decimal places to precisely reflect the estimated effect sizes, regardless of magnitude.

^*^: indicates variables selected for inclusion in the multivariable model based on clinical rationale.

Abbreviations:

CI, confidence interval; Ref, reference;

RBC, red blood cell; WBC, white blood cell; GGT, gamma-glutamyl transpeptidase; ALP, alkaline phosphatase; TBIL, total bilirubin; TP, total protein; HS-CRP, high-sensitivity C-reactive protein; HBDH, hydroxybutyrate dehydrogenase; AST, aspartate aminotransferase; ALT, alanine aminotransferase.
